# Incidence of kidney disease in the canine population treated at a small animal hospital in Uruguay

**DOI:** 10.29374/2527-2179.bjvm013725

**Published:** 2026-08-13

**Authors:** Sofía Perini-Perera, Grazziana Cigliuti-Falero, Paula Pessina, Alejandro Benech-Gulla, Valérie Cayssials

**Affiliations:** 1 Unidad de Clínica y Cirugía de Pequeños Animales, Departamento de Clínicas y Hospital Veterinario, Facultad de Veterinaria, Universidad de la República, Montevideo, Uruguay.; 2 Unidad de Endocrinología y Metabolismo Animal (LEMA), Imagenología y Análisis Clínicos, Departamento Clínicas y Hospital Veterinario, Facultad de Veterinaria, Universidad de la República, Montevideo, Uruguay.; 3 Unidad de Bioestadística, Departamento de Salud Pública, Facultad de Veterinaria, Universidad de la República, Montevideo, Uruguay.

**Keywords:** acute kidney injury, case-control, chronic kidney disease, epidemiology, risk factor, lesão renal aguda, caso-controle, doença renal crônica, epidemiologia, fator de risco

## Abstract

Kidney disease (KD) is a major cause of morbidity and mortality in dogs; however, epidemiological data remain limited. This study estimated the incidence of KD and characterized chronic kidney disease (CKD) and acute kidney injury (AKI) through an observational study of dogs treated at a small animal hospital. A case-control study was subsequently conducted to identify factors associated with KD. Among 2,393 newly registered dogs in 2023, 91 cases of KD were identified, and an equal number of controls were randomly selected. Adjusted odds ratios were estimated using logistic regression models adjusted for age and body condition score (BCS). The overall annual cumulative incidence of KD was 3.8% (95% confidence interval [CI]: 3.0-4.6). The incidence of early-stage CKD was 2.5% (95% CI: 1.9-3.2), followed by advanced-stage CKD at 0.9% (95% CI: 0.5-1.3) and AKI at 0.3% (95% CI: 0.1-0.5). Age was a significant risk factor for KD, with dogs aged ≥10 years having higher odds of KD than younger dogs. Dogs with KD were also more likely to have a poor BCS, and this association was strongest in advanced-stage CKD. To our knowledge, this is the first reported epidemiological study from Latin America to estimate the incidence of KD in dogs. The observed association between KD, advanced age, and poor BCS is based on a detailed review of medical records and the use of appropriate paraclinical investigations, which are essential for early diagnosis and timely intervention to influence the course of the disease.

## Resumo

A doença renal (DR) é uma das principais causas de morbidade e mortalidade em cães; entretanto, os dados epidemiológicos ainda são limitados. Este estudo estimou a incidência de DR e caracterizou a doença renal crônica (DRC) e a lesão renal aguda (LRA) por meio de um estudo observacional de cães atendidos em um hospital veterinário de pequenos animais. Posteriormente, foi realizado um estudo caso-controle para identificar fatores associados à DR. Entre 2,393 cães recém-cadastrados em 2023, foram identificados 91 casos de DR, e um número igual de controles foi selecionado aleatoriamente. As razões de chances ajustadas foram estimadas por meio de modelos de regressão logística ajustados para idade e escore de condição corporal (ECC). A incidência cumulativa anual global de DR foi de 3.8% (intervalo de confiança [IC] de 95%: 3.0-4.6). A incidência de DRC em estágio inicial foi de 2.5% (IC 95%: 1.9-3.2), seguida pela DRC em estágio avançado, com 0.9% (IC 95%: 0.5-1.3), e pela LRA, com 0.3% (IC 95%: 0.1-0.5). A idade foi um fator de risco significativo para DR, com cães com idade ≥10 anos apresentando chances maiores de DR do que cães mais jovens. Cães com DR também apresentaram maior probabilidade de ter um ECC ruim, sendo essa associação mais forte na DRC em estágio avançado. Até onde sabemos, este é o primeiro estudo epidemiológico relatado na América Latina a estimar a incidência de DR em cães. A associação observada entre DR, idade avançada e ECC ruim baseia-se em uma revisão detalhada dos prontuários médicos e no uso de exames complementares apropriados, essenciais para o diagnóstico precoce e a intervenção oportuna capazes de influenciar o curso da doença.

## Introduction

Traditionally, kidney disease (KD) has been classified into chronic kidney disease (CKD), characterized by chronic, progressive, and irreversible structural or functional renal impairment, and acute kidney injury (AKI), a clinical syndrome characterized by a sudden decline in glomerular filtration rate (GFR) resulting from ischemic or nephrotoxic injury (Polzin, 2017). However, recent evidence indicates that these two categories are interconnected, and the distinction between them is often indistinct (Chawla et al., 2014).

KD, including both CKD and AKI, is a significant contributor to morbidity and mortality in dogs (Bedford et al., 2012; Pelander et al., 2015). Despite its clinical relevance, epidemiological data on KD in canine populations remain limited (Babyak et al., 2017). A large-scale study conducted in the United Kingdom reported a CKD prevalence of 0.37% in dogs and identified associations with advanced age and specific breeds (O’Neill et al., 2013). Similarly, a study of 600,000 dogs in Sweden reported a CKD incidence of 1.6% (Pelander et al., 2015). In the United States, Babyak et al. (2017), assessing the prevalence of increased serum creatinine concentration as an indicator of renal damage, reported a prevalence of 11.5%, with increased creatinine concentrations associated with advanced age and neuter status in females.

The availability of local epidemiological data is essential for understanding disease distribution, developing preventive medicine strategies, and improving the prognosis of patients with KD (Bartges, 2012). Delayed diagnosis of KD is associated with disease progression, poor prognosis, and reduced survival (Polzin, 2011). Therefore, determining the incidence of both acute and chronic KD may support the implementation of early diagnostic strategies aimed at slowing disease progression and improving quality of life and survival in affected patients.

To date, no peer-reviewed studies have reported the incidence of KD in veterinary hospitals in Latin America, and the incidence of canine KD in our country remains unknown. Therefore, this study aimed to estimate the incidence of KD and characterize acute and chronic forms of the disease in the canine population treated at the Small Animal Hospital of the Faculty of Veterinary Medicine, Universidad de la República (Udelar). Additionally, we aimed to identify potential associated factors through a case-control study.

## Material and methods

### Study design

An observational study was conducted with the primary descriptive objective of estimating the cumulative incidence of acute and chronic kidney damage among dogs treated at the Small Animal Hospital, Faculty of Veterinary Medicine, Udelar over a 1-year period (January-December 2023). The digitized medical records of all newly admitted canine patients evaluated during this period (n = 2,393) were reviewed. Based on these records, a retrospective longitudinal case-control study was performed to identify clinical cases and evaluate factors associated with KD.

An electronic spreadsheet was manually created to systematically collect and organize data on the variables of interest from each patient's medical record. The reason for consultation at the initial visit was recorded, together with demographic variables used to characterize the study population, paraclinical variables, and potential factors associated with KD. The variables collected included age, sex, reproductive status (intact or neutered), body condition score (BCS; 1-9 scale), hematologic parameters (hematocrit and erythrocyte count), serum biochemical parameters (creatinine and urea concentrations), urinary parameters (urine specific gravity and urine protein-to-creatinine ratio), diet type, and diagnoses of kidney damage and comorbidities documented in each patient's medical record. BSC was categorized according to the World Small Animal Veterinary Association (2025) as poor (BCS 1-3), good (BCS 4-5), or increased (BCS 6-9). Diet type was classified into four categories: (1) high-quality commercial diet, defined as a commercial diet with high nutrient bioavailability and animal protein sources of high biological value; (2) low-quality commercial diet, characterized by lower nutrient bioavailability due to poor-quality protein sources; (3) home-prepared diet, consisting of fresh ingredients formulated by the owner; and (4) mixed diet, comprising a combination of commercial diet and home-prepared diet. Comorbidities were recorded for each patient and categorized according to the affected medical specialty: endocrinology, gastroenterology, cardiology, dermatology, oncology, odontology, nephrology, neurology, traumatology, or other.

A total of 91 dogs were classified as cases. All had a documented clinical history of acute or chronic KD, supported by medical records and paraclinical findings indicative of structural and/or functional kidney damage. Diagnostic criteria included evidence of structural damage (e.g., ultrasonographic findings such as loss of corticomedullary definition, renal infarction, surface irregularities, or cortical hyperechogenicity) and/or functional impairment, defined by elevated GFR markers (e.g., serum creatinine (>1.4 mg/dL) or a documented history of persistently decreased fasting urine specific gravity (≤1.030).

Cases were classified into five groups according to the International Renal Interest Society (IRIS) staging guidelines (International Renal Interest Society, 2025). Group 1 included dogs with evidence of mild chronic kidney damage, characterized by persistently decreased urine specific gravity, proteinuria, or structural renal abnormalities identified by ultrasonography, but without increased GFR markers, determined by the absence of elevated serum creatinine (<1.4 mg/dL). Group 2 included dogs with mildly elevated GFR markers (serum creatinine, 1.4-2.8 mg/dL). Group 3 included dogs with moderate azotemia, defined by a serum creatinine concentration of 2.9-5.0 mg/dL. Group 4 included dogs with severely decreased GFR, defined by a serum creatinine concentration >5.0 mg/dL. Group 5 included dogs diagnosed with AKI based on the medical record, accompanied by elevated serum creatinine concentrations and clinical signs consistent with uremic syndrome. For statistical analyses, Groups 1 and 2 were categorized as early-stage CKD, whereas Groups 3 and 4 were categorized as advanced-stage CKD.

The control group comprised 91 dogs, matching the number of study cases. Control dogs were randomly selected from patients newly admitted to the hospital during the same year. Eligible dogs had no history of KD, GFR markers within the species-specific reference intervals, a urine specific gravity (USG) ≥1.030, and no ultrasonographic evidence of structural kidney damage. Patients were excluded if they had incomplete data required to assess KD, such as missing renal profile results or abdominal ultrasonography, or if they had concurrent diseases associated with acute alterations in blood volume (e.g., decompensated cardiac or metabolic disease). Dogs receiving glucocorticoid therapy were also excluded. In addition, dogs with prerenal azotemia, defined as increased serum urea and creatinine concentrations in the presence of a USG >1.030, were excluded.

### Statistical analysis

The cumulative annual incidence of KD, with corresponding 95% confidence intervals (95% CIs), was estimated overall and by CKD stage, including patients with AKI (Group 5). Incidence was calculated as the proportion of KD cases among all patients newly admitted to the hospital in 2023.

Case and control characteristics were summarized using the median and interquartile range (IQR) for quantitative variables (age, hematologic parameters, biochemical parameters and urinary parameters) and frequencies with percentages for qualitative variables. Differences between cases and controls were assessed using the Mann-Whitney *U* test for continuous variables and the chi-square test of independence for categorical variables (sex, reproductive status, body condition, and diet). Hematologic, biochemical and urinary parameters were compared among the control, early-stage CKD, advanced-stage CKD, and AKI groups using the Kruskal-Wallis test.

Variables that were statistically significant in the univariate analyses were included in a multivariate logistic regression model to estimate adjusted odds ratios and corresponding 95% CIs. The contribution of each remaining variable to the final model was evaluated using likelihood ratio tests, comparing models with and without the variable of interest. To determine whether factors associated with KD differed between early-stage and advanced-stage CKD, separate logistic regression analyses were performed for each subgroup.

All statistical analyses were conducted using jamovi version 2.3, with statistical significance defined as a *p*-value <0.05.

## Results

A total of 3,279 medical records of newly admitted patients were reviewed in 2023, of which 2,393 corresponded to dogs. Among these records, 91 canine patients had clinical signs of chronic or acute KD confirmed by diagnostic testing. Of these cases, 39.6% (36/91) were classified as Group 1, 27.5% (25/91) as Group 2, 11.0% (10/91) as Group 3, and 13.2% (12/91) as Group 4. AKI was diagnosed in 8.8% (8/91) of the affected patients.

The overall annual cumulative incidence of KD was 3.8% (95% CI, 3.0-4.6). The cumulative incidence was 2.5% (95% CI, 1.9-3.2) for early-stage CKD, 0.9% (95% CI, 0.5-1.3) for advanced-stage CKD, and 0.3% (95% CI, 0.1-0.5) for AKI.

Among the 91 dogs diagnosed with KD, the median age was 10 years (IQR, 8-12 years), compared with 8 years in the control group. Age differed significantly between cases and controls (Mann-Whitney *U* test, *p* <0.001).

No significant differences were observed in sex distribution between cases and controls. Females accounted for 61.5% of cases and 62.6% of controls, with similar proportions of males in both groups. Likewise, reproductive status (intact or neutered) did not differ significantly between groups (Table 1). In contrast, body condition differed significantly (*p* = 0.005), with poor body condition being more common among dogs with KD than among controls. No significant differences were observed in diet between the two groups (Table 1).

**Table 1:** Demographic characteristics of patients with chronic or acute kidney disease (cases) and patients without kidney disease (controls) at the Small Animal Hospital of the Faculty of Veterinary Medicine, Universidad de la República (Udelar), in 2023.

**Variable**	**Case**	**Control**	**Chi-square test**
	**n = 91**	**n = 91**	***p-*value**
Age (years), n (%)			
≤6	16 (17.6)	34 (37.3)	χ^2^ = 14.4, df = 2; *p* < 0.001
7-9	20 (22.0)	27 (29.7)	
≥10	54 (59.3)	30 (33.0)	
No data	1 (1.1)	-	
Sex, n (%)			
Female	56 (61.5)	57 (62.6)	
Male	35 (38.5)	34 (37.4)	χ^2^ = 0.023, df = 1; *p* = 0.88
Reproductive status, n (%)			
Intact	30 (33.0)	33 (36.3)	χ^2^ = 0.129, df = 1; *p* = 0.72
Neutered	58 (63.7)	57 (62.6)	
No data	2 (2.2)	1 (1.1)	
Body condition, n (%)			
Poor	28 (30.8)	12 (13.2)	χ^2^ = 10.5, df = 2; *p* = 0.005
Good	31 (34.1)	47 (51.6)	
Increased	14 (15.4)	20 (22.9)	
No data	18 (19.8)	12 (13.2)	
Diet, n (%)			
High-quality CD	28 (30.8)	31 (34.1)	χ^2^ = 2.82, df = 3; *p* = 0.42
Low-quality CD	13 (14.3)	23 (25.3)	
Home-prepared diet	14 (15.4)	11 (12.1)	
Mixed diet	20 (22.0)	19 (20.9)	
No data	16 (17.6)	7 (7.7)	

Abbreviations: CD, commercial diet.

The most common breeds among cases were Poodles and Labrador Retrievers (7.7% each; 7/91), followed by Golden Retrievers (5.5%; 5/91) and mixed-breed dogs (51.7%; 47/91). In the control group, the most represented breeds were Poodles (8.8%; 8/91), Labrador Retrievers (5.5%; 5/91), Dachshunds (4.4%; 4/91), Golden Retrievers (4.4%; 4/91), and mixed-breed dogs (47.3%; 43/91).

**Figure 1:**
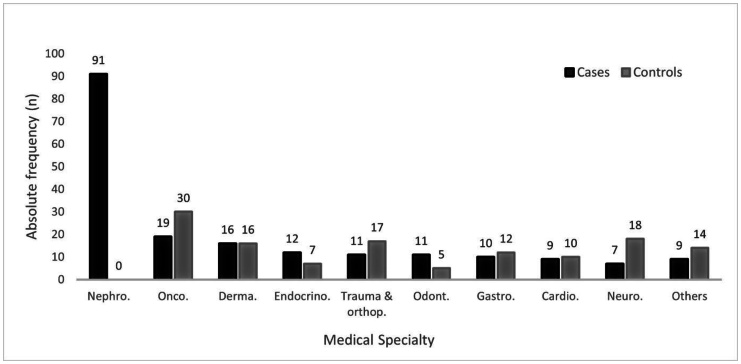
Absolute frequencies of diagnosed comorbidities by consultation specialty.

Regarding comorbidities, 25.3% (23/91) of dogs with KD had no additional comorbidities, 41.8% (38/91) had one, 24.2% (22/91) had two, and 8.8% (8/91) had three or more. In the control group, 61.5% (56/91) presented with a single clinical complaint or affected organ system, 35.2% (32/91) presented with two, and 3.3% (3/91) presented with three or more. Among dogs with KD, the most frequent comorbidities were oncological diseases (20.9%; 19/91), followed by dermatological (17.6%; 16/91), endocrine (13.2%; 12/91), traumatological and orthopedic (12.1%; 11/91), dental (12.1%; 11/91), gastroenterological (11.0%; 10/91), cardiovascular (9.9%; 9/91), neurological (7.7%; 7/91), and other diseases (9.9%; 9/91) (Figure 1).

**Figure 2:**
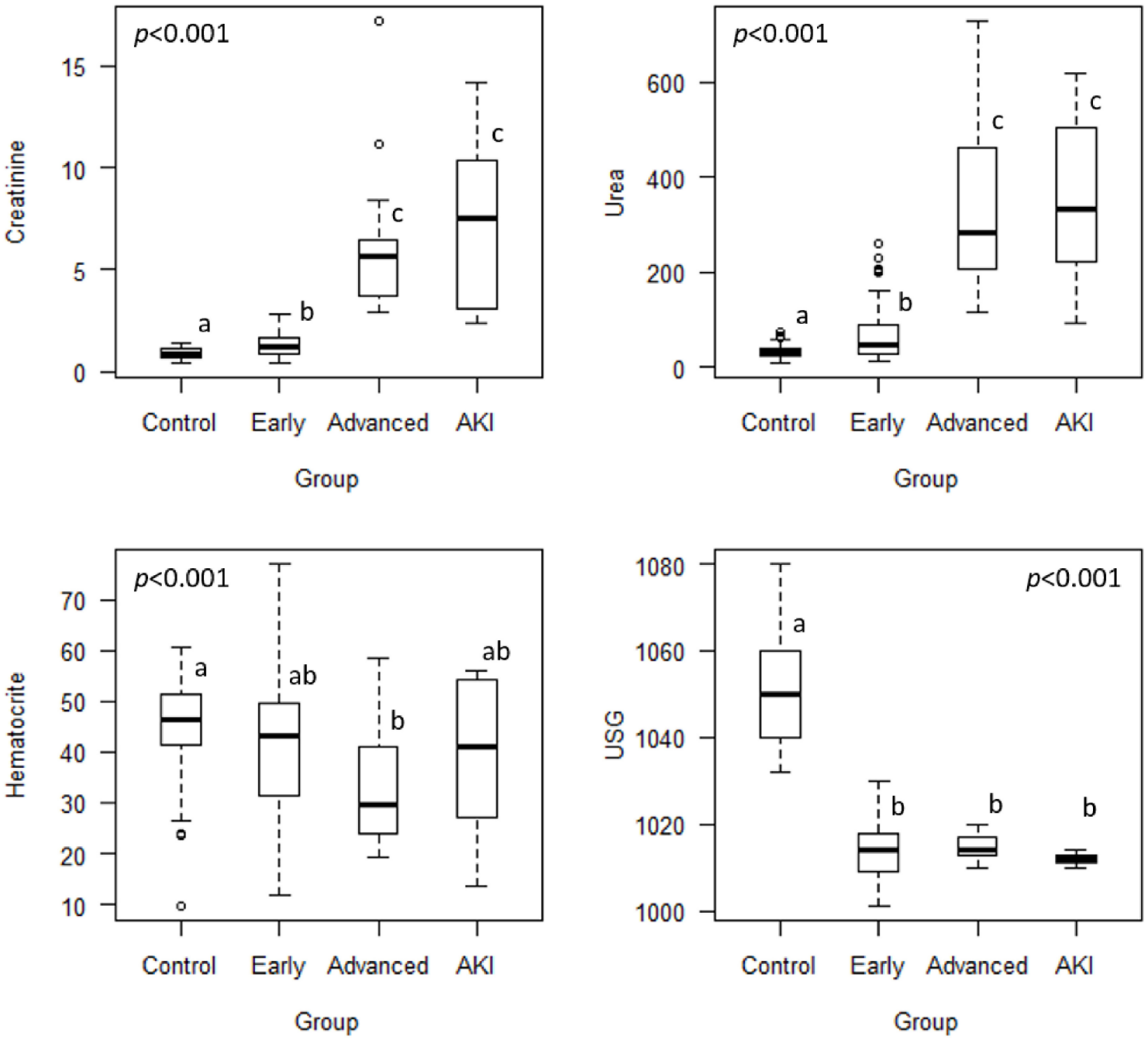
Hematological, biochemical and urinary parameters (creatinine, urea, hematocrit, and USG) in dogs categorized by kidney disease stage (controls, early-stage CKD, advanced-stage CKD, and AKI). Statistical comparisons among groups were performed using the nonparametric Kruskal-Wallis test. Abbreviations: AKI, acute kidney injury; CKD, chronic kidney disease; USG, urine specific gravity. Note: Different letters indicate statistically significant differences (*p* <0.05) in pairwise comparisons following *post hoc* analysis. Early-stage CKD included Groups 1 and 2, while advanced-stage CKD included Groups 3 and 4.

Table 2 summarizes the hematological, biochemical and urinary parameters of dogs with KD and the control group. Significant differences were observed in hematocrit, urea, creatinine, and USG between cases and controls. Figure 2 shows the distribution of these diagnostic biomarkers and the results of the Kruskal-Wallis test comparing the control, early CKD, advanced CKD, and AKI groups. *Post hoc* analyses showed that serum creatinine and urea differed significantly between the control group and the early CKD, advanced CKD, and AKI groups. Hematocrit differed significantly only between the control group and the advanced CKD and AKI groups. USG was significantly lower in all KD groups (early CKD, advanced CKD, and AKI) than in the control group.

**Table 2:** Concentration of hematological, biochemical and urinary parameters in patients with chronic or acute kidney disease (cases) and those without kidney disease (controls) at the Small Animal Hospital of the Faculty of Veterinary Medicine, Universidad de la República (Udelar), in 2023. The *p*-values from the Mann-Whitney *U* test used for comparisons between groups are presented.

**Biomarker** (units)	*n*	**Case**	*n*	**Control**	***p*-value**
		median (Q1-Q3)		median (Q1-Q3)	
Erythrocytes (mill/µl)	88	5.9 (4.6-7.5)	90	6.8 (5.9-7.6)	0.005
Hematocrit (%)	88	40.1 (27.8-48.9)	90	46.3 (41.4-51.2)	<0.001
Urea (mg/dL)	89	93.1 (35.0-231)	91	32.2 (24.3-38.9)	<0.001
Creatinine (mg/dL)	90	1.7 (1.10-3.58	91	0.90 (0.70-1.10)	<0.001
USG (g/ml)	50	1.014 (1.010-1.018)	25	1.050 (1.040-1.060)	<0.001
UPC	13	1.70 (0.30-2.00)	4	0.50 (0.39-0.52)	0.16

Abbreviations: Q1, first quartile; Q3, third quartile; UPC, urine protein-to-creatinine ratio; USG, urine specific gravity. Note: Due to the small sample size, the UPC comparison lacks sufficient statistical power to draw definitive conclusions.

Table 3 presents the crude and adjusted odds ratios for the variables significantly associated with KD: age and body condition. After age stratification, dogs ≥10 years old had more than fivefold higher odds of KD than dogs ≤6 years old (adjusted odds ratio [OR_adj_] = 5.3; 95% CI, 2.2-12.8). After adjustment for age, dogs with KD had fourfold higher odds of poor body condition than controls (OR_adj_ = 4.0; 95% CI, 1.7-9.7). Similar results were observed in subgroup analyses of early and advanced CKD, with a stronger association between poor body condition and advanced CKD.

**Table 3:** Crude and adjusted odds ratios for age and body condition estimated using univariate and multivariate logistic regression models among all kidney disease cases and subgroups stratified by early- and advanced-stage CKD.

**Variable**	**crude OR**	**[95% CI]**	***p*-value**	**adjusted OR**	**[95% CI]**	***p*-value**
**All KD**						
Age (years)						
≤6	1 (ref.)			1 (ref.)		
7-9	1.6	0.7-3.6	0.28	1.7	0.6-4.5	0.30
≥10	3.8	1.8-8.0	<0.001	5.3	2.2-12.8	<0.001
Body condition						
Good	1 (ref.)			1 (ref.)		
Poor	3.5	1.6-8.0	0.002	4.0	1.7-9.7	0.002
Increased	1.1	0.5-2.4	0.89	1.0	0.4-2.4	0.98
						
**Early-stage CKD**						
Age (years)						
≤6	1 (ref.)			1 (ref.)		
7-9	2.2	0.9-5.8	0.10	2.2	0.8-6.7	0.14
≥10	4.5	1.9-10.9	<0.001	5.8	2.1-15.9	<0.001
Body condition						
Good	1 (ref.)			1 (ref.)		
Poor	2.4	0.96-5.8	0.06	2.7	1.0-7.2	0.04
Increased	1.1	0.5-2.7	0.79	1.1	0.4-2.7	0.87
						
**Advanced-stage CKD**						
Age (years)						
≤6	1 (ref.)			1 (ref.)		
7-9	0.8	0.1-5.4	0.85	0.6	0.1-8.1	0.72
≥10	6.8	1.8-25.4	0.004	11.9	1.9-72.9	0.007
Body condition						
Good	1 (ref.)			1 (ref.)		
Poor	7.1	2.0-25.0	0.002	11.2	2.4-53.0	0.002
Increased	0.9	0.2-5.3	0.94	0.9	0.1-5.4	0.89

Abbreviations: CI, confidence interval; CKD, chronic kidney disease; KD, kidney disease; OR, odds ratio; ref., reference. Note: Early-stage CKD included Groups 1 and 2, while advanced-stage CKD included Groups 3 and 4.

## Discussion

### Incidence

This study found a cumulative annual incidence of KD of 3.8% (95% CI: 3.0-4.6), which is approximately three times higher than the incidence reported in a Swedish study (1.6%) that used a large canine database (Pelander et al., 2015). In contrast, two studies from India employing designs similar to ours reported incidences of 2.1% (Karunanithy et al., 2021) and 2.6% (Tufani et al., 2015). Despite these differences, the incidence observed in our study is of the same order of magnitude as those reported previously. The variability in incidence estimates may reflect differences in study populations, hospital settings, and case inclusion criteria. In our study, a comprehensive census of all newly admitted patients was conducted using elevated serum creatinine concentrations and a clinical history of kidney damage as inclusion criteria. By contrast, the Swedish study identified cases solely based on a documented diagnosis of KD in electronic medical records, without applying standardized diagnostic criteria. This approach may have underestimated the true incidence of KD because it relied on physician-recorded diagnoses, which are subject to differences in clinical experience and diagnostic practices across hospitals (Pelander et al., 2015).

Several studies have also reported the prevalence of KD, with estimates ranging from 0.37% (O’Neill et al., 2013) to 11.5% (Babyak et al., 2017) and 11.9% (Sosnar et al., 2003). These marked differences are also likely attributable to variations in case ascertainment methods. O’Neill et al. (2013) identified cases based on clinician-recorded diagnoses in electronic medical records and the presence of relevant keywords, whereas Babyak et al. (2017) and Sosnar et al. (2003) defined KD solely on the basis of serum creatinine concentrations, irrespective of the clinician's diagnosis. In the present study, medical records were reviewed manually, and serum creatinine concentrations were evaluated without relying on diagnostic keywords or recorded diagnoses. This approach likely increased the sensitivity of case detection compared with methods based exclusively on electronic medical record coding or keyword searches (O’Neill et al., 2013).

### Study groups

The distribution of CKD stages observed in our study was similar to that reported in a previous study conducted in Mexico (Perini-Perera et al., 2021), with a higher proportion of patients in the early stages of CKD and fewer cases in advanced stages. In contrast, studies conducted in the United States and England reported a greater proportion of patients with advanced CKD (Babyak et al., 2017; O’Neill et al., 2013). These differences may be attributable to variations in case inclusion criteria. In the present study, medical records were systematically reviewed for early indicators of CKD, including persistently decreased urine specific gravity, abnormalities identified on diagnostic imaging, and proteinuria, to establish a diagnosis of CKD. By contrast, the other studies relied solely on the presence of azotemia (Babyak et al., 2017) or database keyword searches to identify eligible cases (O’Neill et al., 2013).

### Risk factors associated with KD

#### Age

The median age of dogs in the present study was 10 years, consistent with previous studies of canine KD. Sosnar et al. (2003) reported a mean age of 8.7 years, Perini-Perera et al. (2021) reported a median age of 10 years, and O’Neill et al. (2013) reported a mean age exceeding 12 years. These findings are consistent with the well-established association between aging and KD. In older dogs, functional renal mass progressively declines because of age-related nephron loss and cumulative renal injury from causes such as nephrotoxicity and ischemia, making KD a common condition in this population (Bartges, 2012). In contrast, Pelander et al. (2015) reported a lower mean age at diagnosis (6.9 years). This difference may be attributable to the study's selection criteria, which included only insured dogs up to 10 or 12 years of age, thereby excluding older animals and potentially underestimating the age at disease presentation.

Age-stratified analysis showed that dogs aged ≥10 years had a significantly higher risk of KD, consistent with the findings of O’Neill et al. (2013) and Babyak et al. (2017). CKD was the most common form of KD identified in the present study. Because CKD predominantly affects older dogs (Bartges, 2012), its high prevalence likely contributed to the significant age-related difference observed between cases and controls.

#### Sex and reproductive status

No significant associations were identified between sex or reproductive status and KD in the animals evaluated, consistent with previous reports (O’Neill et al., 2013; Pelander et al., 2015). However, this finding contrasts with that of Babyak et al. (2017), who reported that spayed females are at increased risk of KD. This discrepancy may be explained by perioperative renal injury associated with surgical anesthesia, which is generally more prolonged and complex in females than in males, potentially increasing the risk of ischemic damage (Fossum, 2019). Alternatively, the association may be confounded by pyometra, a recognized risk factor for renal injury that the retrospective design of the study could not adequately assess (Maddens et al., 2011).

#### Body condition

This study identified a significant association between KD and poor body condition. Cases in the early stages of CKD had nearly three times the risk of having poor body condition compared with age-matched controls. Similarly, patients in advanced CKD stages had a higher risk of poor body condition than controls. These findings are consistent with those of a previous study, which reported a higher proportion of patients with reduced body condition in advanced CKD stages (Perini-Perera et al., 2021). Poor body condition in CKD is multifactorial. Contributing factors include reduced food intake resulting from appetite loss, uremic gastroenteritis, and age-related sarcopenia (Parker, 2021; Ross, 2011). In addition, CKD is associated with uremic sarcopenia, which is driven by metabolic and hormonal abnormalities characteristic of the disease, including dyslipidemia, thyroid dysfunction, metabolic acidosis, accumulation of uremic toxins, disturbances in calcium-phosphorus metabolism, and vitamin D deficiency. Chronic inflammation also contributes to muscle loss through increased circulating concentrations of inflammatory mediators, including interleukins (e.g., IL-6 and IL-8), tumor necrosis factor, interferon-γ, and other cytokines (Bakinowska et al., 2024; Mohanasundaram & Fernando, 2022). Assessment of body condition is therefore clinically important in patients with KD because loss of muscle mass reduces serum creatinine concentrations, potentially leading to overestimation of the true GFR in patients with poor body condition (Hall et al., 2015).

#### Diet

The type of diet offered did not differ significantly between cases and controls, with a comparable distribution of dietary categories observed in both groups. Only three cases were receiving a renal prescription diet at the time of consultation, a dietary intervention known to slow the progression of CKD (Hall et al., 2018). However, the retrospective design of this study limited the availability of additional dietary information. Furthermore, the dietary categories evaluated (high- and low-quality commercial diets, home-prepared diets, and mixed diets) did not consider specific dietary criteria associated with KD.

#### Breed

The breeds most frequently affected were mixed-breed dogs, Poodles, and Labrador Retrievers. This finding is consistent with previous reports, in which Golden Retrievers, Schnauzers, and Chihuahuas were also identified as commonly affected breeds (Pérez-Sánchez et al., 2023). Similarly, a study conducted in India reported the Labrador Retrievers as the most frequently affected breed (Tufani et al., 2015). Conversely, Bernese Mountain Dogs, English Cocker Spaniels, Doberman Pinschers, Dalmatians, and Rottweilers have been reported as breeds commonly affected by KD, likely due to their predisposition to glomerular injury (Pelander et al., 2015); however, these findings differ from those observed in the present study. Such discrepancies, as well as the overrepresentation of mixed-breed dogs and certain breeds within the study population, may be attributed to breed distribution and preferences in the geographic region evaluated, particularly given the similar breed composition observed between cases and controls.

#### Comorbidities

The most frequently observed comorbidities in this study were oncological, dermatological, and endocrine disorders. These findings may be related to the reported association between these conditions and the development of glomerular structural alterations, including damage to the basement membrane, endothelium, and podocytes. Such changes can increase glomerular permeability, allowing the passage of larger proteins and resulting in proteinuria (Littman et al., 2013). Because neoplasms have been associated with the development of proteinuria, they are considered a potential risk factor for CKD, highlighting the importance of routine proteinuria assessment in cancer patients for early detection of renal impairment (Prudic et al., 2018). Similarly, endocrine disorders such as hyperadrenocorticism may exert detrimental effects on renal function by inducing hemodynamic changes and promoting glomerular and tubular dysfunction (Smets et al., 2010). The findings of the present study differ from those reported by O’Neill et al. (2013), who identified stronger associations between KD and periodontal disease, cardiac disease, and musculoskeletal disorders. However, this discrepancy may be explained by referral hospital bias, as these three specialties represent a substantial proportion of newly admitted patients at the institution included in the present study. Consequently, the prevalence of KD among patients with these conditions may be overestimated (Bartlett et al., 2010).

#### Hematological and biochemical parameters

The statistical differences observed between cases and controls in serum creatinine and urea concentrations are consistent with the criteria used to define cases and reflect the decrease in GFR that occurs in patients with KD. The differences observed among early CKD stages, advanced CKD stages, and AKI are likely associated with progressively greater declines in renal function in the latter groups, which are characteristic of advanced CKD and clinical-stage AKI (Polzin, 2011). Similarly, the lower USG observed in the KD groups compared with the control group is consistent with the impaired ability to concentrate urine that occurs in these conditions as a result of compromised tubular function (Heiene & Lefebvre, 2007).

Finally, hematocrit differed between the control group and the advanced CKD group, with the latter showing median values below the expected range for the species. This finding may be associated with multiple factors that contribute to the development of anemia in patients with advanced CKD (Chalhoub et al., 2011). These factors include decreased erythropoietin production, increased erythrocyte fragility associated with uremia and secondary renal hyperparathyroidism, and gastrointestinal bleeding resulting from ulcers and platelet dysfunction (King et al., 1992).

### Study limitations

Several limitations should be considered when interpreting the findings of this study. First, selection bias may have affected both the case and control groups, as inclusion was based on pathological laboratory findings and medical history. Consequently, patients with KD who lacked laboratory test results or had incomplete medical records may have been excluded, potentially leading to an underestimation of the cumulative incidence observed.

Regarding the statistical robustness of the analyses, the substantial amount of missing data for the BCS variable in the case-control analysis may have reduced statistical power and limited the generalizability of the findings. Furthermore, the small sample size resulted in wide confidence intervals in the subgroup analysis of advanced CKD stages, thereby limiting the precision, statistical power, and reliability of these estimates.

The inclusion of early markers of CKD, such as symmetric dimethylarginine, could have improved the sensitivity of detecting patients in the early stages of the disease. Additionally, classification into azotemic groups (Groups 2, 3, 4, and AKI) was based on the first creatinine measurement available, which may have limited the ability to accurately determine disease chronicity or distinguish acute from chronic renal pathology. To address this limitation, medical records were thoroughly reviewed for clinical indicators of chronicity (e.g., polyuria, polydipsia, weight loss, anorexia, hyporexia, and halitosis) and chronic renal structural abnormalities identified by ultrasound. These findings were used to support case classification and improve differentiation between acute and chronic cases.

## Conclusion

To our knowledge, this is the first epidemiological study conducted in Latin America to estimate the incidence of KD in dogs. We identified an association between KD and poor body condition, as well as advanced age. The differences observed compared with previous studies highlight the importance of a comprehensive evaluation of medical history and the use of appropriate paraclinical diagnostic tests to facilitate early diagnosis and potentially influence the progression of KD.
